# Use of honey associated with *Ananas comosus* (Bromelin) in the treatment of acute irritative cough

**DOI:** 10.1016/j.rppede.2016.04.002

**Published:** 2016

**Authors:** Décio Medeiros Peixoto, José Angelo Rizzo, Deborah Schor, Almerinda Rêgo Silva, Dinaldo Cavalcanti de Oliveira, Dirceu Solé, Emanuel Sarinho

**Affiliations:** a Universidade Federal de Pernambuco (UFPE), Recife, PE, Brazil; b Universidade Federal de São Paulo (Unifesp), São Paulo, SP, Brazil

**Keywords:** Cough, Honey, Bromelin

## Abstract

**Objective:**

To evaluate the immediate improvement rate of irritative cough in patients treated with the combination of *Ananas comosus* extract and honey (Bromelin^®^) compared with the use of honey alone (placebo group).

**Methods:**

Pragmatic, double-blind, randomized, parallel-group study with children aged between 2 and 15 years, with irritative cough for at least 24hours. The double-blind assessment of cough was through the number of observed coughing episodes and intensity score for a period of 10minutes of observation. The decrease of one point in the mean total score was considered as a therapeutic effect.

**Results:**

There was a reduction in coughing episodes in both groups, as well as in the cough score after 30minutes of drug or honey administration. The change in clinical score above two points, which could indicate marked improvement, occurred in five patients in the bromelin group and only in one in the placebo group, but without significant difference. There were no adverse events.

**Conclusions:**

The immediate improvement rate of irritative cough was similar in patients treated with combination of *Ananas comosus* extract and honey (Bromelin^®^) compared with the use of honey alone (placebo group). It is possible that honey has a therapeutic effect on mucus and cough characteristics (Clinical Trials: NCT01356693).

## Resumo

**Objetivo:**

Avaliar a taxa de melhoria imediata da tosse irritativa em pacientes tratados com associação do extrato do *Ananas comosus* em mel de abelha (Bromelin^®^) e compará-la com a de uso do mel isolado (grupo placebo).

**Métodos:**

Estudo pragmático da vida real, duplo-cego, randômico, de grupos paralelos, em crianças, entre dois e 15 anos, com tosse irritativa havia pelo menos 24 horas. A avaliação da tosse foi duplo-cega foi feita por meio da avaliação do número de episódios observáveis e do escore de intensidade de tosse durante o período de 10 minutos de observação. A redução de um ponto na média do escore total foi considerada como efeito terapêutico.

**Resultados:**

Em ambos os grupos houve redução do número de episódios de tosse, assim como do escore de tosse após 30 minutos de administração do medicamento ou do mel. A mudança de escore clínico superior a dois, que poderia indicar melhoria acentuada, ocorreu em cinco pacientes do grupo com bromelina e em apenas um do placebo, mas sem diferença significante. Não ocorreram eventos adversos.

**Conclusões:**

A taxa de melhoria imediata da tosse irritativa foi similar entre pacientes tratados com associação do extrato do *Ananas comosus* em mel de abelha (Bromelin^®^) e com o uso do mel isolado (grupo placebo). É possível que haja um efeito terapêutico do mel nas características do muco e da tosse (Clinical Trials: NCT01356693).

## Introduction

Viral processes of the airways are usually self-limited and characterized mainly by fever and coughing, which are two independent predictors of the virus presence and tend to disappear in approximately one week. However, coughing is the most common symptom in primary care physicians' offices and is a frequent cause of visits to emergency care; this symptom can become so intense to the point of leading patient and relatives to exhaustion.^1^^-^ 3

In such cases, symptomatic drugs are often used by the patients that self-medicate, often in response to advertisement and the fact that they do not require a prescription. Available in pharmacies and drugstores, with different formulations, substances for cough relief can bring risks, especially in the pediatric age group.^3^^,^ 4

Dextromethorphan and diphenhydramine are often used as antitussive agents, especially in children.^5^ A clinical study compared these two therapeutic principles with honey, regarding the control of nocturnal cough and sleep quality of children with irritative cough, and found that honey (2.5mL) administered before sleep, showed better results regarding symptom relief when compared with these two drugs.^6^ A recent Cochrane Library review, carried out with clinical studies with acceptable methodological quality standards, did not report any evidence for or against the effectiveness of these drugs in the symptomatic relief of patients with cough.^4^ Therefore, an ideal therapeutic strategy for symptomatic control of cough remains uncertain to date.^3^^-^ 5

Consequently, the individual and the relatives distressed by the coughing seek measures they consider safe for cough relief, when this symptom is intense. In this aspect, empirical therapy based on natural and/or herbal products, has been increasingly employed and many people use it freely, with or without medical prescription, secondary to the relatives' suggestion and/or the patient's own beliefs.^3^

In this context, products derived from *Ananas comosus* extract (rich in Bromelin) and honey have shown promising results, as they contain proteolytic enzymes such as trypsin, chymotrypsin and Bromelin.^7^ Bromelin is the generic name for the family of proteolytic enzymes containing the sulfhydryl radical derived from *A. comosus*, the pineapple plant, which also has nonproteolytic enzymes, with significant topical action in the respiratory mucosa.^7^^,^ 8 Free of toxic effects, these pineapple-derived products have shown promising properties in *in vitro* studies: they interfere with the growth of malignant cells, inhibit platelet aggregation and coagulation and have mainly an anti-inflammatory and mucolytic action. These are potentially beneficial actions; Bromelin has shown to have a significant action in dissolving bronchial secretion, with the advantage of being a food constituent and, thus, a safe substance for use by children.^9^^-^ 13

The World Health Organization indicates honey as a potential treatment of cough in upper respiratory tract infections in children, being considered a symptomatic, inexpensive, popular and safe treatment. Studies have attributed antioxidant, antibacterial, antifungal, antiviral, anti-inflammatory and antitumor actions to honey, as well as immunomodulatory properties.^14^^-^ 16 A double-blind, randomized trial evaluated the effectiveness of three types of honey (eucalyptus, citrus and labiatae) in the control of nocturnal cough and quality of sleep in children with upper respiratory tract infections and compared them with placebo. The three active treatment regimens with honey showed significant improvement of the assessed parameters when compared to the previous night.^15^

The combination of *A. comosus* extract with honey may represent a breakthrough in the symptomatic treatment, possibly by blocking the triggering mechanisms of cough due to viral or irritative conditions. Thus, this study was aimed to evaluate the effect on the immediate inhibition of irritative cough in patients treated with a combination of *A. comosus* (pineapple) and honey (Bromelin^®^) and compare them with those treated with honey alone (placebo) in a pediatric emergency care environment.

## Method

This pragmatic, real-life, double-blind, randomized study of parallel groups was carried out in the pediatric service of Clinica Amaury Coutinho, linked to Recife City Hall. Patients in the active group (letter A) received the combination of honey and *A. comosus* extract HBS19820501 (rich in Bromelin) as syrup formulation and the placebo group (letter B) received only honey. The quality of the honey was certified in both groups and approved by the regulatory agencies and the National Health Surveillance Agency (Anvisa) for sale; the honey had been recently produced and underwent strict bacteriological control. Randomization was carried out according to a table generated by Excel program. The double-blinding was released and the treatment groups were revealed only after the analysis of the study results.

Children aged between two and 15 years, with irritative cough for at least 24hours, which led to the need for medical consultation, participated in the study. We excluded patients with a history of obstructive pulmonary disease, cystic fibrosis, neuropathies, heart disease, diabetes and identifiable primary or secondary immunodeficiencies. Patients should have acute cough due to viral upper airway infection, thus considered due to the presence of mild fever or fever associated with hyaline or catarrhal rhinorrhea, lasting less than 72hours, without clinical manifestations of associated bronchospasm. After meeting the inclusion criteria, patients were randomly assigned to a treatment group (Bromelin^®^ or honey). The patients' parents and/or guardians agreed with their participation and signed the informed consent form. The two treatment regimens were administered as follows: (a) children up to 20kg received 5mL; (b) for those weighing>20kg, 1mL was administered for every five kilos of additional weight. Before the randomization, patients with detectable bronchospasm were prescribed β-2 adrenergic and, if necessary, a single-dose of oral steroids and the patient was considered ineligible for the study. The organoleptic characteristics of Bromelin^®^ and honey are similar.

Patients' evolution and clinical data after treatment were reported in a standardized clinical report form. Cough assessment was carried out in double-blind fashion by one of the investigators by counting the number of episodes observed and the cough intensity score during a 10-min observation period. The initial and final cough intensity score was classified according to a tool as 0=absent; 1=mild, 2=moderate; 3=intense; 4=very intense, according to a previous study.^5^ According to this tool, a decrease of one point in the mean total score is considered as therapeutic effect. After waiting 30minutes post-drug administration, the therapeutic response was assessed during a 10-minute observation period, which was chosen as it is feasible and satisfactory in the actual clinical setting of an emergency care service.

Therefore, after the coughing episodes observed by the researcher were quantified in the 10minutes prior to treatment administration, the standardized observation was repeated 30minutes after drug administration, again in a double-blind fashion. All evaluations before and after the drug or placebo administration were carried out by a single and the same investigator (physician) in a double-blind fashion (neither the investigator nor the patient, nor the family knew which product was used), in order to attain robust results.

The medication safety was assessed by the frequency of reported adverse events, such as episodes of vomiting, epigastric and abdominal pain, among others. The monitoring of subsequent adverse events was not performed.

To calculate sample size, the following was considered: power of test of 90%, rate of cough improvement in the active group of 40%, and 10% in the placebo group, two-tailed hypothesis test and 5% significance level. Thus, 60 patients were necessary to assess the possible therapeutic effects of the active treatment. This calculation is according to the clinical trial by Muller et al., who studied approximately 30 patients per group to assess the possible effect of Bromelin on inflammatory markers, but with the consequent impact on the clinical improvement of cough.^8^

Statistical analysis took into account the nature of the assessed variables. Numerical variables were expressed as mean and standard deviation or, when necessary, in medians and 25-75 percentiles, whereas categorical ones were expressed as percentages. Numerical variables were compared using Student's *t* test, whereas the chi-square test was used for categorical ones. The value was considered significant when *p*<5%.

The study was approved by the Institutional Review Board of Centro de Ciências da Saúde da Universidade Federal de Pernambuco, with CAAE n. 0311.0.172.000-10 and the trial was registered at Clinical Trials sob under n. NCT01356693 (*ClinialTrials.gov Identifier*).

## Results

Table 1 demonstrates that the two groups of patients were similar in age, gender, days of coughing and mean clinical score of coughing episodes. Table 2 shows that both groups had a reduction in coughing episodes, as well as in the cough score 30minutes after drug or honey administration. However, after analyzing the change in clinical score higher than two points, which could indicate a more accentuated improvement, it was seen to have occurred in five patients in the active group and one in the placebo group. There were no immediate adverse events.

**Table 1:** General baseline characteristics of the Bromelin and Placebo (honey) study groups regarding gender, mean age, days of symptom persistence and initial cough severity score.

	Group		*p*-value
	Bromelin^®^ (n=31)	Placebo (n=29)	
*Gender M/F*	13/18	13/16	0.97^a^
*Age in years-mean (P25-P75)*	5.3 (3.0-7.5)	5.6 (2.0-7.5)	0.82^b^
*Days of symptoms*			0.98^a^
1	6	6	
2	10	9	
3	15	14	
*Coughing episodes-median (P25-P75)*	3 (2-3)	3 (2-4)	0.98^b^
*Cough score-median*	2	2	0.91^b^
*Score>2, n (%)*	26 (84%)	26 (90%)	0.87^a^

Days of symptoms, number of days with symptoms of cough before consultation; coughing episodes-median (P25-P75), number of coughing episodes during the 10minutes of initial observation by the doctor; cough score-median, initial cough score observed by the doctor (0=absent; 1=mild; 2=moderate; 3=intense; 4=very intense).

a Chi-square.

b Wilcoxon Test.

**Table 2:** Change in the number of coughing episodes and cough scores between the observation periods (10minutes) before and 30minutes after administration of the treatment regimen in 60 evaluated patients.

	Group		*p*-value
	Bromelin^®^ (n=31)	Placebo (n=29)	
*Coughing episodes*			
Before [Median (P25-P75)]	3 (2-3)	3 (2-4)	
After [Median (P25-P75)]	1 (1-1)	1 (1-1)	
Before-after difference (Mean±SD)	1.71±0.78	1.76±0.87	0.83^a^
*Reduction in coughing episodes after medication (n)*			0.94^b^
0 (no change)	2	2	
1	11	9	
2	13	12	
3	4	6	
4	1	0	
Before-after difference (Mean±SD)	-0.97±0.62	-0.86±0.45	0.32^a^
*Variation in the clinical cough score after the medication*			0.22^b^
0 (no change)	6	6	
-1	30	32	
-2	5	1	

a Student's *t* test.

b Chi-square.

## Discussion

When the bronchial mucosa is injured by chemical, physical or infectious agents, it responds with an inflammatory process, in which epithelial cells start to produce excessive amounts of mucus.^17^ After a viral infection occurs, there is an increase in the release of cytokines, neurotransmitters and leukotrienes, which induce an increase in neural receptor levels, with transient stimulation of afferent neural activity, mucus hypersecretion and, possibly, exacerbation of the effects on cholinergic motor pathways. The feeling of irritation that precedes the cough motor action allows us to infer the concept of cough syndrome due to hypersensitivity after acute respiratory viral infection, which, in some patients, becomes a refractory, exhausting cough due to inflammation and excess mucus.^17^ Although diphenhydramine and dextromethorphan are the most commonly used drugs in the management of irritative cough, they have not been shown to be superior to placebo in controlling this symptom.^18^ On the other hand, a comparative study between dextromethorphan and buckwheat honey showed their equivalence, with honey superiority when compared with placebo-treated patients.^15^

The mucolytic action of certain drugs can promote reduction in mucus viscosity and facilitate its elimination by coughing and through ciliary beating in the bronchial epithelium. This is possible due to the breakdown of mucoproteins, which splits them into polypeptides. Furthermore, the anti-inflammatory action of a specific drug can contribute to the reduction in thick mucus production.^7^^,^ 17 ^,^ 19 The extract of *A. comosus*, present in the product used in the present study, contains proteolytic enzymes (Bromelin, ribonuclease, glucose oxidase, invertase and diastase) and bivalent cations of trace elements (magnesium, manganese, zinc, iron and calcium) that act as cofactors for the functions of these enzymes. The enzymes catalyze the breakdown of peptide bonds by incorporating water molecules, thus facilitating the fluidization of thick mucus.^7^^,^ 19

*In vitro* studies indicate that the anti-inflammatory activity of Bromelin, results in part from the inhibition of bradykinin formation at the inflammation site, through the depletion of plasma kallikrein levels, as well as the reduction of intermediates of the coagulation cascade, which limits the formation of fibrin. Additionally, Bromelin reduces leukocyte migration, as well as the expression of adhesion molecules (CD128) to blood vessels.^7^^,^ 19 ^,^ 20 The possible beneficial effect of Bromelin may be due to an integrated series of actions on cells and cytokines, such as: natural killer (NK) cell activation, increased production of tumor necrosis alpha factor, as well as Interferon-gamma (IFN-γ) several interleukins (IL-1, IL-2, IL-6) and of granulocyte-macrophage colony-stimulating factor and seems capable of interrupting the continuous activation of CD4+ lymphocytes, which maintain the inflammatory process.^21^

Additionally, in healthy humans, oral ingestion of Bromelin promotes a change in the circadian cycle of IFN-γ, IL-5 and IL-10, suggesting an immunomodulatory effect on T lymphocytes.^21^ This observation has been the rationale for the use of Bromelin, either alone or associated with other natural products in the treatment of autoimmune and inflammatory diseases, such as ulcerative colitis, multiple sclerosis and respiratory tract disorders.^21^^,^ 22 In children with acute sinusitis, Bromelin was added to the standard therapy and there was a significant reduction in symptom duration and time of recovery, when compared with those who received only the conventional treatment; it is extremely safe, with only a mild and self-limiting allergic reaction in a patient allergic to pineapple.^13^

Honey was the common constituent of the two forms of treatment, which leads us to believe that the mucolytic and cough relief properties of honey were responsible for the reduction in the cough score, as well as the reduction in the number of coughing episodes observed in the two groups of assessed patients.^15^^,^ 23 The literature has suggested that honey may be indicated as a rational measure in the treatment of cough, as it is low cost, has rare adverse events and there is some evidence of a possible therapeutic action.^16^^,^ 24

This clinical trial suggests that, considering the patent improvement in both groups, there probably is a therapeutic effect of honey on mucus and cough characteristics, which may have made it difficult to identify differences when honey is associated with the possible additional pharmacological and favorable action of Bromelin. The suggested hypothesis is that the sweetness of honey itself promotes salivation and secretion production in the airways and leads to mucus liquefaction, reducing cough due to the lower irritation in the larynx and pharynx and, most importantly, with no side effects, so it can be safely used in children older than one year, when the intestinal microbiome is structured and defined and there is adequate immunity against *Clostridium botulinum*, a possible contaminant of honey.^16^^,^ 25 ^,^ 26 In a multicenter trial carried out in Europe, honey was used in children aged 1 to 18 years without any side effects and with possible therapeutic benefits.^27^

The present study, as mentioned before, is a pragmatic clinical trial carried out in a real-life scenario of an emergency service, which may have created some limitations, such as the need to use more objective and reproducible indicators to assess cough improvement, especially in a population of children with such a wide age range wide for the analysis of clinical sign caused by such diverse pathologies. However, all the study patients had acute cough associated with upper airway infection and, additionally, all evaluations were carried out in double-blind fashion and standardized by a single researcher, allowing an internal validation of the study. An evaluation method using video, a longer time of observation and a better-defined outline of existing diseases would be ideal, but would hinder the operational assessment in a representative clinical scenario of emergency care.

The addition of Bromelin to honey does not result in an additional effect in the treatment of irritative cough. It seems that a possible effect of Bromelin, if any, would be better assessed by using another substance as placebo, because honey can be a pharmacologically active substance in coughing.^25^^-^ 27 Similarly, new clinical trials with a longer observation period, assessing other outcomes are necessary to evaluate this potential therapeutic effect of honey, either alone or associated, on irritative cough. In daily clinical practice, parents, relatives and patients are interested in this explanation with some urgency, considering their care and safety and the possibility of relief for the annoyance that irritating cough causes to patients.^16^^,^ 25 ^,^ 26
